# Metamaterials for Wearable Textile Antennas: Materials, Structures, and Fabrication

**DOI:** 10.3390/ma19163398

**Published:** 2026-08-10

**Authors:** Ruihua Wang, Qingyun Tao, Yong Zhang, Jiyong Hu

**Affiliations:** 1 College of Textiles, Donghua University, Songjiang Campus, 2999 North Renmin Road, Shanghai 201620, China; wrhdhu@163.com (R.W.);; 2 School of Textiles and Fashion, Jiangsu College of Engineering and Technology, Chongchuan Campus, 87 Qingnian Middle Road, Nantong 226006, China

**Keywords:** metamaterials, metasurfaces, textile antennas, wearable antennas, on-body performance

## Abstract

With the rapid development of wearable body-centric wireless systems, there is a growing demand for textile antennas with stable on-body performance, low profile, flexibility, and garment compatibility. Textile metamaterials provide an effective approach to address the limitations of conventional textile antennas by regulating antenna–body coupling, backward radiation, surface-wave propagation, frequency selectivity, local resonance, and polarization. Although existing reviews have discussed the mechanisms and structures of metamaterials, systematic discussions that connect metamaterial structures with textile materials, fabrication, and performance remain limited. This review summarizes representative metamaterials used in textile antennas and analyzes their roles in gain enhancement, SAR reduction, miniaturization, multiband operation, and polarization improvement. Common substrates, spacers, and conductive materials are further reviewed, together with fabrication methods such as lamination, embroidery, sewing, weaving, knitting, printing, coating, and laser patterning. Current studies indicate that material variability, conductor loss, fabrication tolerance, layer alignment, deformation stability, and garment integration still restrict practical application. This review is expected to provide a reference for the design and realization of reliable metamaterials for textile antennas.

## 1. Introduction

Wearable textile antennas are key radio-frequency components for body-centric wireless systems, where conformability, low weight, flexibility, and user comfort must be achieved together with stable electromagnetic performance [1,2,3,4,5,6]. A central difficulty in such antennas is their operation in the lossy near-body environment. Human tissues can perturb the input impedance, shift the resonant frequency, reduce gain and radiation efficiency, enhance backward radiation, and raise specific absorption rate (SAR) concerns [1,5]. This problem becomes more complex on textile platforms, since textile substrates generally exhibit low permittivity, porous morphology, and moisture-sensitive dielectric properties due to their hygroscopic nature, while conductive yarns and fabrics introduce finite conductivity, sheet resistance, and process-dependent current paths [3,7,8]. Metamaterials are artificially engineered structures designed to manipulate electromagnetic waves beyond the capability of natural materials. They offer an effective route to regulate antenna–body coupling and preserve radiation performance by tailoring reflection phase, surface impedance, wave propagation, frequency selectivity, polarization response, and electromagnetic absorption [9,10].

Different metamaterial structures have been introduced as functional layers or resonant elements in textile antenna systems. High-impedance surfaces (HIS) and artificial magnetic conductors (AMC) provide in-phase reflection and are commonly used as low-profile backing layers for suppressing body-side radiation; textile AMC-backed antennas have demonstrated improvements in gain, efficiency, and SAR performance [9,10,11,12]. Electromagnetic band-gap (EBG) structures mainly act through surface-wave and coupling suppression, leading to enhanced front-to-back ratio, radiation efficiency, and on-body stability; representative textile EBG antennas have been reported to exhibit reduced body-directed radiation, gain enhancement, and SAR reduction [13,14,15,16]. Frequency-selective surfaces (FSS) function as selective reflectors, shielding layers, filters, or superstrates, and have been used for gain enhancement, SAR reduction, beam control, and frequency-selective shielding in textile platforms [17,18,19,20,21]. In addition, metamaterial-inspired resonators, including split-ring resonators (SRR), complementary split-ring resonators (CSRR), composite right/left-handed (CRLH) cells, and zeroth-order resonators (ZOR), provide local resonance mechanisms for miniaturization, multiband operation, and bandwidth tuning [22,23,24,25,26,27]. Polarization-control metamaterials further support polarization conversion and circular-polarization (CP) improvement [28,29,30,31,32,33], whereas absorptive metamaterial structures introduce controlled loss for leakage-field attenuation, electromagnetic shielding, and exposure reduction [34,35,36,37]. These studies show that metamaterials are not simply auxiliary loading elements, but electromagnetic design tools for coordinating the antenna, textile platform, human body, and wireless channel.

**Figure 1:**
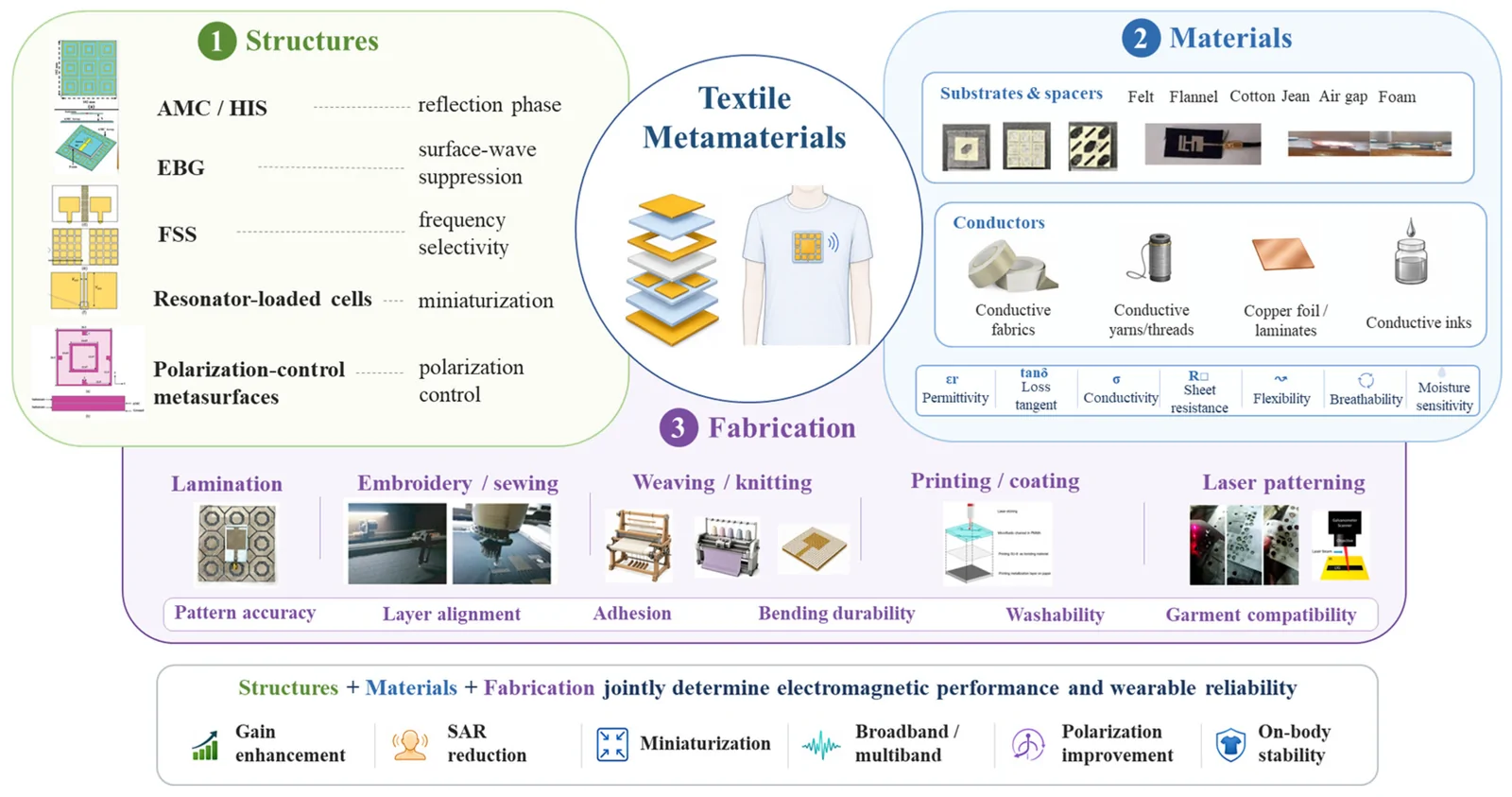
Schematic overview of the structures, materials, fabrication, and performance of metamaterial-integrated wearable textile antennas. (AI-assisted tools were used to provide preliminary suggestions for the layout and color scheme and to generate several small schematic icons. The final figure was manually reconstructed, reviewed, and edited by the authors using Microsoft PowerPoint, version 16.0 (Microsoft Corporation, Redmond, WA, USA)).

Previous reviews have separately addressed wearable and textile antennas in terms of antenna configurations, textile materials, fabrication, and human-body effects [3,4,5,6,38,39,40,41], and have also summarized the electromagnetic mechanisms and functions of metamaterials [10,16,42]. However, these discussions often emphasize metamaterial structures, while the textile realization of metamaterials remains less connected to materials and fabrication. This review therefore connects metamaterial structures, textile materials, textile-compatible fabrication, and performance. As summarized in Figure 1, the review of metamaterials for wearable textile antennas can be discussed from three closely related aspects: structures, materials, and fabrication, with emphasis on antenna–body coupling control, radiation improvement, SAR reduction, polarization regulation, and garment integration.

Relevant studies were retrieved from Web of Science, Scopus, IEEE Xplore, ScienceDirect, and Google Scholar, covering publications from [1990] to [2026]. Search terms included combinations of “textile antenna”, “wearable antenna”, “textile metamaterial”, “AMC”, “HIS”, “EBG”, “FSS”, and “metasurface”. Studies on textile, flexible, or wearable metamaterial structures for antenna integration were included, particularly those reporting simulation, measurement, material, or fabrication information. Studies limited to rigid, optical, or terahertz structures without clear relevance to wearable textile antennas were excluded. Titles and abstracts were screened first, followed by full-text assessment.

## 2. Metamaterial Structures and Mechanisms in Textile Antennas

### 2.1. AMC and HIS

AMC and HIS are reflective metamaterial structures mainly defined by high surface impedance and a near-zero reflection phase around resonance. This feature distinguishes it from EBG and FSS structures. EBG mainly emphasizes the suppression or control of surface-wave propagation, while FSS regulates frequency-selective transmission or reflection. AMC/HIS is therefore more closely related to the replacement or modification of a metallic reflector, allowing the radiator to operate with a smaller spacing from the backing layer [9,10].

Structurally, textile AMC/HIS designs are usually composed of periodic conductive units on soft dielectric substrates. Common cells include square patches, rings, loops, grids, octagonal units, and C-shaped resonators. The gaps between adjacent units mainly provide equivalent capacitance, while the conductive paths and substrate thickness contribute to inductance. Conductive fabrics, plated textiles, embroidered conductors, and copper-based fabrics are often combined with felt, foam, denim, cotton, or other textile-compatible substrates to form flexible impedance surfaces [11,12,43,44,45,46,47].

The placement of the AMC/HIS layer determines how strongly it interacts with the radiator. In many wearable antennas, the AMC/HIS is used as a body-side backing layer and is separated from the radiator by foam, felt, or an air gap. This configuration mainly redirects backward radiation, improves the front-to-back ratio and gain, and reduces SAR. For example, Yan et al. used a low-profile dual-band AMC to stabilize the textile antenna response, while Ali et al. adopted a 3 × 3 dual-band AMC array for gain, efficiency, bandwidth, and SAR improvement [11,12]. El Atrash et al. further demonstrated that AMC-backed wearable antennas can combine gain enhancement with low-SAR operation in WBAN-oriented designs [48,49].

In another configuration, the AMC/HIS is placed close to the radiator or integrated into the antenna stack. In this case, it is no longer only a reflector; it also participates in impedance tuning, cavity formation, polarization control, or resonant loading. Bait-Suwailam et al. used a textile HIS to improve the impedance of a grounded loop antenna, showing that HIS layers are not limited to patch-type antennas [50]. Joshi et al. used a 3 × 3 square-patch AMC backing for a dual-band, dual-sense textile antenna, linking the AMC layer with GPS localization and WBAN/WLAN operation [51]. Yang et al. further showed that a polarization-rotation AMC can support dual-band dual-circularly polarized off-body communication [52].

For textile patch antennas with a continuous ground, placing an AMC/HIS below the ground should be considered carefully. If the ground is electrically continuous, most body-side fields are already shielded, and the AMC/HIS may couple weakly with the radiator. This layout becomes more meaningful when the ground is partial, slotted, windowed, or grid-like, where leakage toward the body still exists. In such cases, an additional AMC/HIS reflector or a breathable grid-type AMC can provide extra isolation while partly preserving flexibility and air permeability, as illustrated by the breathable ground-grid AMC reported by Hussain et al. [53].

Table 1 summarizes representative AMC/HIS structures reported in wearable textile antennas, with emphasis on unit-cell form, integration position, and the corresponding antenna-level functions. Overall, AMC/HIS design in textile antennas is not only a matter of selecting a unit-cell shape. It requires coordinated control of cell geometry, array size, layer spacing, substrate thickness, conductor continuity, bending stability, and garment comfort. These structural factors determine whether the AMC/HIS behaves as an effective body-side reflector, an embedded impedance surface, or a weakly coupled auxiliary layer.

### 2.2. EBG

EBG structures are periodic surfaces that create a stopband for electromagnetic-wave propagation. In wearable textile antennas, they are mainly used to suppress or control surface waves near the antenna–textile–body interface. Unlike AMC/HIS, which is usually associated with high surface impedance and in-phase reflection, and FSS, which focuses on frequency-selective transmission or reflection, EBG structures are more directly related to band-gap behavior, surface-current suppression, and coupling control [9,10,16].

Textile EBGs are commonly formed by periodic conductive cells on flexible substrates. Patch-type, coplanar, dual-band, and via-less layouts are frequently adopted. Since metallic vias are difficult to fabricate and maintain in soft fabrics, via-less EBGs are especially suitable for textile antennas, although their stopband response is sensitive to unit-cell size, gap spacing, substrate thickness, conductor continuity, and bending deformation [13,14,16].

Representative studies show the main functions of EBG in textile antennas. Zhu and Langley used a dual-band EBG to reduce body-side radiation and improve gain [13]. Ashyap et al. demonstrated that a via-less textile EBG can enhance gain, efficiency, front-to-back ratio, and SAR performance without using vertical vias [14]. Wissem et al. extended textile EBG design to a 26 GHz 5G-IoT antenna, while Agus et al. and Kumkhet et al. applied EBG-based surfaces to wearable MIMO systems for isolation, radiation improvement, and SAR reduction [15,58,59]. In addition, Tak et al. showed that EBGs can also guide useful body-surface waves rather than only suppress unwanted propagation [60].

Table 2 summarizes representative EBG structures in wearable textile antennas, including dual-band EBGs, via-less EBGs, millimeter-wave EBG-backed antennas, and EBG-assisted MIMO designs. These examples indicate that textile EBGs are mainly used for surface-wave control, body-side isolation, gain and efficiency enhancement, SAR reduction, and mutual-coupling suppression. Overall, textile EBG design requires a compromise between electromagnetic performance and textile compatibility. A stable band gap depends on periodicity and layer accuracy, while bending, compression, cutting tolerance, and misalignment may shift the operating frequency or weaken the stopband. Therefore, EBG-based textile antennas should be evaluated together with gain, efficiency, SAR, front-to-back ratio, bending stability, and on-body performance.

### 2.3. FSS

FSS are periodic conductive or aperture-type surfaces that provide frequency-dependent transmission and reflection responses. In wearable textile antennas, FSS are mainly used as selective reflectors, shielding layers, or superstrates. Compared with continuous metallic grounds, textile FSSs can offer electromagnetic isolation while retaining better flexibility, lower weight, and partial breathability. This makes them suitable for gain enhancement, SAR reduction, radiation-pattern control, and body-side shielding in garment-integrated antennas [17,18,19,20].

Textile FSS are usually formed by repeated unit cells such as square loops, rings, grids, cross-shaped elements, Y-shaped fractal cells, or other patterned conductive fabrics. Their response is determined by unit-cell geometry, periodicity, substrate permittivity, conductor sheet resistance, and the spacing between the FSS and radiator. In textile implementations, fabrication tolerance is particularly important because cutting error, lamination direction, embroidery roughness, bending, and fabric deformation can shift the resonant frequency or weaken the frequency-selective response [18,19,20,21].

Representative antenna studies show the main role of FSSs in wearable textile platforms. Amit et al. used an FSS reflector beneath a textile antenna to improve gain and reduce SAR at 2.4 and 5.8 GHz [17,61]. Sugumaran et al. reported a compact flexible monopole backed by a modified square-loop FSS for SAR reduction and gain enhancement [62]. Vaezi et al. further combined an FSS with a miniaturized circularly polarized wearable antenna, where the FSS contributed to beam shaping and SAR control [63]. In addition, frequency-selective fabric studies by Rac-Rumijowska et al., Guan et al., Li et al., and Zheng et al. show that textile substrate, unit-cell shape, lamination direction, and fabrication method strongly affect FSS transmission and shielding performance [18,21,64,65,66,67,68].

Table 3 summarizes representative FSS and frequency-selective textile structures used in wearable textile antennas. The listed studies include textile FSS reflectors, square-ring FSSs, ring-shaped and Y-shaped frequency-selective fabrics, and FSS-based coupling-control structures. These examples indicate that FSSs are mainly used for gain enhancement, SAR reduction, beam shaping, electromagnetic shielding, transmission control, and coupling suppression. Overall, FSS design in textile antennas requires a balance between frequency selectivity and wearable compatibility. A denser or larger FSS array can improve shielding and radiation control, but it may also increase stiffness and reduce comfort. Meanwhile, bending, compression, and textile anisotropy can alter the unit-cell spacing and effective permittivity. Therefore, FSS-based textile antennas should be evaluated not only by free-space transmission or reflection response, but also by antenna gain, SAR, radiation pattern, bending stability, and on-body performance.

Table 4 provides a concise category-level comparison of the three main metamaterial structures used in wearable textile antennas, clearly highlighting their differences in structural features, primary functions, and application focuses.

### 2.4. Representative Performance Visualizations

Previous sections summarized the quantitative performance variations in textile metamaterial antennas. This section further illustrates the effects of metamaterial integration on radiation, impedance matching, and human safety through visual comparisons.

**Figure 2:**
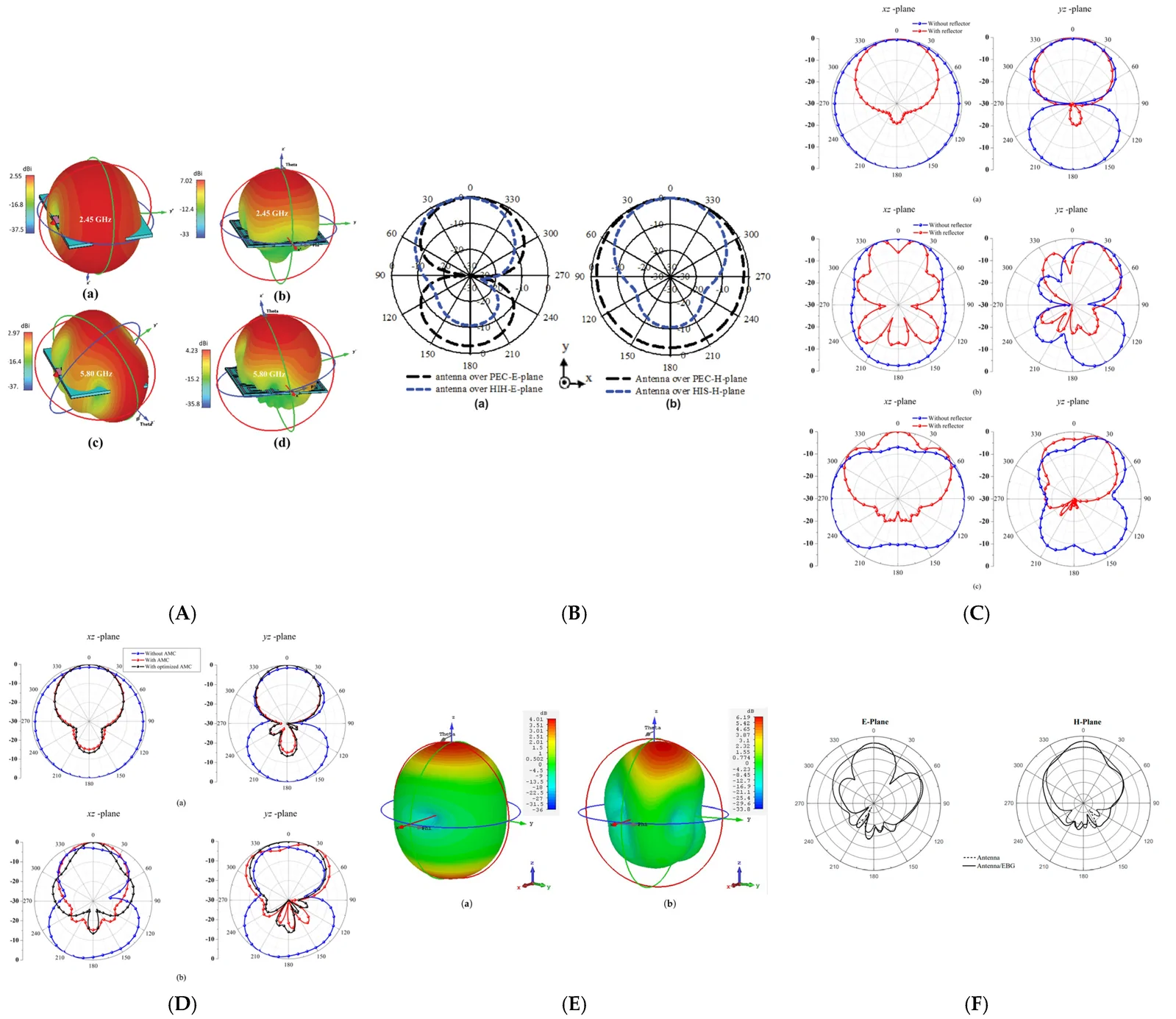
Representative radiation-pattern variations in wearable textile antennas before and after metamaterial integration: (**A**) [12] Ali; (**B**) [43] Ashyap; (**C**) [45] Thaiwirot; (**D**) [46] Hengroemyat; (**E**) [50] Bait-Suwailam; (**F**) [15] Wissem.

As shown in Figure 2, most textile antennas exhibit reduced backward radiation and enhanced forward gain upon integration of metamaterial structures [12,15,43,45,46,50]. AMC/HIS structures primarily redistribute electromagnetic energy through reflection-phase control, reducing body-directed radiation, while EBG structures suppress surface-wave propagation and reduce unwanted energy losses.

**Figure 3:**
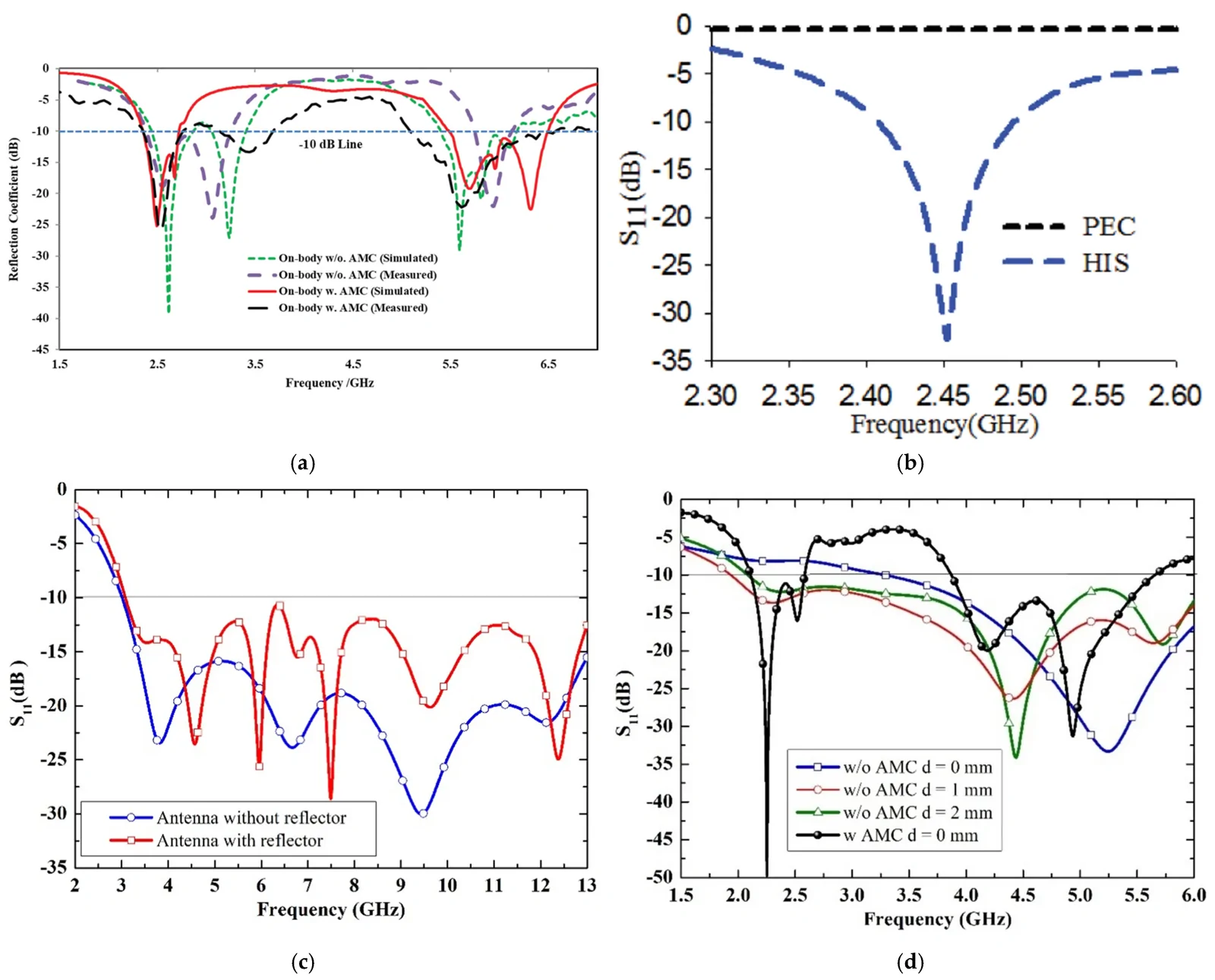
Representative S11 variations in wearable textile antennas before and after metamaterial integration: (**a**) [12] Ali; (**b**) [43] Ashyap; (**c**) [45] Thaiwirot; (**d**) [46] Hengroemyat.

Figure 3 shows that metamaterial loading can improve the impedance matching performance of textile antennas. The results reported by Ashyap [43] and Hengroemyat [46] demonstrate bandwidth enhancement and reduced reflection loss after AMC integration. However, Ali [12] and Thaiwirot [45] indicate that metamaterial loading may also modify the equivalent inductance, capacitance, and resonant modes of the antenna, resulting in operating-frequency shifts that require further frequency tuning.

**Figure 4:**
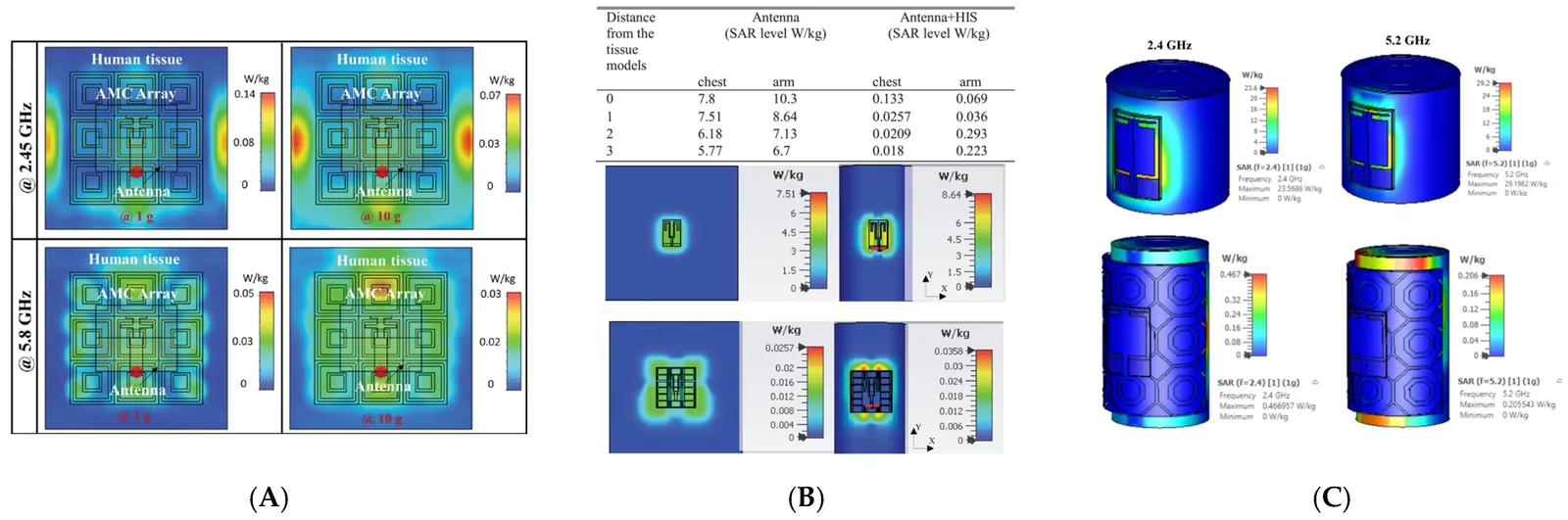
Representative SAR variations in wearable textile antennas before and after metamaterial integration: (**A**) [12] Ali; (**B**) [43] Ashyap; (**C**) [46] Hengroemyat.

As shown in Figure 4, SAR reduction is one of the major motivations for integrating metamaterials into wearable antennas. AMC/HIS and EBG structures effectively reduce electromagnetic leakage toward the human body. However, additional reflective layers and spacer structures may increase antenna profile and compromise flexibility and garment integration.

Overall, textile metamaterials provide effective approaches for improving radiation performance and human safety, but their advantages involve trade-offs with wearable compatibility. Furthermore, many existing designs still rely on concepts developed for rigid substrates, highlighting the need for textile-oriented metamaterial designs that consider the unique characteristics of textile materials, including flexibility, deformation, and anisotropy.

In addition to AMC, HIS, EBG, and FSS structures, metamaterial-inspired resonators and polarization-control metasurfaces provide localized electromagnetic control in wearable textile antennas. Resonator-loaded elements, such as SRRs, CSRRs, CRLH cells, ZORs, and anisotropic metasurface cells, can be embedded into the radiator, feed line, ground, slot region, or nearby textile layer to introduce additional resonances, adjust current paths, and tune impedance, bandwidth, miniaturization, or multiband performance [22,23,24,25,26,27,70,71]. Polarization-control metasurfaces are especially useful for wearable communication, GNSS, and positioning systems because they can improve circular polarization, axial-ratio bandwidth, gain stability, and off-body communication under changing antenna orientations [28,29,30,31,32,33,72,73,74]. Therefore, metamaterial structures in textile antennas should be evaluated not only by their structural type, but also by their placement, coupling strength, and antenna-level functions, including matching, radiation pattern, gain, efficiency, SAR, polarization response, deformation stability, and fabrication repeatability [9,10,16,17,18,19,20,21].

## 3. Materials for Metamaterial Structures in Textile Antennas

### 3.1. Substrates and Spacer Materials

Substrates and spacer layers strongly affect the electromagnetic response of textile metamaterial antennas. The substrate determines the effective permittivity, loss, unit-cell capacitance, and electrical size, while the spacer controls the coupling strength between the radiator, the metamaterial layer, and the human body. A higher permittivity can reduce the unit-cell size, but excessive dielectric loss may lower radiation efficiency. A thicker spacer can improve isolation and broaden the effective reflection response, but it also increases antenna profile and reduces wearing comfort [9,10,16].

Felt is the most widely used substrate in textile AMC and EBG antennas because it is soft, low-cost, low-permittivity, and easy to obtain in millimeter-scale thickness. It has been used in dual-band EBG/HIS antennas, AMC-backed textile antennas, and recent all-textile AMC designs [11,12,13,45,46]. These studies show that substrate thickness, intercell gap, and conductive-cell geometry jointly determine the reflection phase, band-gap position, and antenna–body isolation. Foam, Rohacell, air gaps, and spacer fabrics are often added to adjust the separation between the radiator and the metamaterial layer, thereby improving gain, front-to-back ratio, and SAR performance [13,17,46,48].

FSS and polarization-control metasurfaces introduce broader substrate choices, including flannel, polyester textile, PET fabric, and multilayer felt. For example, Amit et al. used flannel with an air gap in an FSS-backed textile antenna, while Rac-Rumijowska et al. showed that substrate permittivity, thickness, conductive layer properties, and interelement spacing all influence FSS frequency selectivity [17,18]. These results indicate that textile substrates should be treated as electromagnetic design variables rather than only as garment materials.

Table 5 summarizes representative substrate and spacer materials used in textile metamaterial antennas, including felt, flannel, foam, Rohacell, air gaps, PET textile, cotton, jean, jute, PDMS, and Ecoflex. The listed parameters show that material thickness, relative permittivity, and loss tangent vary significantly across reported designs, which partly explains the differences in resonance shift, reflection bandwidth, SAR reduction, and bending stability.

Overall, substrate and spacer selection requires a compromise among miniaturization, bandwidth, isolation, comfort, and mechanical stability. Future studies should report not only nominal dielectric parameters but also the effects of compression, humidity, bending, and washing effects, since these factors can change the effective permittivity, layer spacing, and final antenna performance.

### 3.2. Conductive Materials

Conductive materials define the current path of textile metamaterial unit cells. In AMC/HIS, EBG, FSS, metasurfaces, and resonator-loaded antennas, conductor properties affect surface resistance, current continuity, ohmic loss, intercell coupling, and fabrication accuracy. Therefore, the conductor is not only a metallization layer, but also a key factor controlling reflection phase, stopband response, frequency selectivity, absorption strength, and radiation efficiency.

Table 6 summarizes representative conductive materials used in textile metamaterial antennas and related structures. The reported choices include copper- or nickel-plated conductive fabrics, ShieldIt-type textiles, copper foil or polyimide copper laminates, conductive threads, printed silver inks, PU resistive films, conductive fibers, liquid metal, MXene-coated fabrics, and conductive 3D-printing filaments. Their electrical properties vary widely, from highly conductive copper layers to resistive films and fibrous networks designed for controlled loss.

Conductive fabrics are still the most common option because they are flexible, easy to cut or laminate, and sufficiently conductive for many wearable antenna bands [11,12,13,14,45,46]. However, their performance depends strongly on sheet resistance, edge quality, adhesive bonding, and bending stability. Fraying, anisotropic conductivity, and local air gaps may shift resonance or reduce radiation efficiency. At higher frequencies or for finer metasurface patterns, conductor precision becomes more critical. Copper laminates and foils provide better edge definition and lower loss, which is useful for millimeter-wave EBGs and compact metasurfaces [15,28]. Embroidered conductive threads offer better textile integration and washability, but stitch density, thread tension, and surface roughness can change the effective line width and resistance [24,25,35,78]. For absorbers and tunable structures, the target is not always maximum conductivity; resistive films, conductive fibers, carbon-based coatings, and liquid metals are selected to provide controlled loss or deformation-dependent tuning [34,35,36,37,75,76].

Overall, conductive-material selection should match the electromagnetic function of the structure. Reflective layers usually require low-loss and continuous conductors. Future studies should report not only conductivity, but also sheet resistance, pattern resolution, bending durability, adhesion, washing stability, and their influence on antenna performance.

Material selection and fabrication accuracy are critical for metamaterial-integrated textile antennas because the electromagnetic response is strongly coupled with the textile platform. They directly affect equivalent capacitance, inductance, surface impedance, band-gap behavior, frequency selectivity, absorption loss, and polarization response. Compared with rigid microwave substrates, textile materials introduce additional design uncertainty. Their porosity, compressibility, anisotropy, moisture sensitivity, and deformation under bending or washing can shift the resonant frequency, change the reflection or transmission response, and reduce repeatability. Similarly, conductive fabrics, threads, printed inks, liquid metals, and lossy coatings differ in conductivity, sheet resistance, pattern resolution, adhesion, and durability, which further influence radiation efficiency, SAR reduction, shielding performance, and long-term wearable reliability.

## 4. Metamaterials Fabrication and Integration Technologies

### 4.1. Fabric Lamination

Fabric lamination is one of the most common fabrication routes for textile metamaterial antennas. Conductive fabrics, copper foils, or copper tapes are cut into periodic unit cells and then bonded onto felt, foam, denim, polyester, or other flexible substrates. This method is especially suitable for AMC/HIS, EBG, and FSS structures, where the electromagnetic response depends strongly on unit-cell size, gap spacing, and layer alignment [13,14,43,44,45,46,48,50].

Table 7 summarizes representative laminated textile metamaterial antennas, including patterned conductive-fabric HIS, low-profile AMC layers, octagonal AMC cells, textile HIS-backed antennas, and metamaterial-inspired laminated patterns.

Lamination is a simple and low-cost method for rapid prototyping of textile metamaterial antennas. Conductive fabrics or copper foils can be cut into periodic patterns and bonded onto flexible substrates, making it convenient to verify designs, compare unit-cell geometries, adjust spacer layers, and assemble multilayer antenna–metamaterial stacks [45,46,53,55]. However, lamination is not a high-precision textile manufacturing method. Cutting errors, adhesive thickness, bonding nonuniformity, and layer misalignment may alter the effective substrate height, intercell capacitance, reflection phase, stopband position, and impedance matching. Adhesive bonding may also increase local stiffness, reduce breathability, or cause delamination during repeated deformation. Therefore, laminated textile metamaterial antennas are suitable for laboratory-stage validation, but practical garment integration still requires evaluation of dimensional repeatability, bonding stability, flexibility, and long-term reliability.

### 4.2. Embroidery and Sewing

Embroidery and sewing are widely used for textile antennas because conductive threads can be patterned into radiators, resonators, and cells. They are especially useful for SRR-based antennas, RFID reader antennas, and textile resonant structures [24,25,35,66,67,78,83].

Table 8 summarizes representative embroidered or sewn textile metamaterial structures. These studies show that embroidery is not only a manufacturing method but also a way to tune electromagnetic response through stitch density, thread path, and conductive-thread continuity. The electromagnetic mechanism is strongly linked to stitch geometry. Stitch density, stitch direction, yarn position, yarn tension, and stabilizer choice control the effective line width, surface roughness, inter-yarn contact resistance, and continuity of each unit cell. These parameters alter the equivalent inductance and capacitance of the design [35,84,85].

The advantage of embroidery is its compatibility with large-area textile production and washable garment integration. At the same time, the process introduces RF uncertainty. Stitch spacing, yarn tension, thread roughness, and inter-yarn contact resistance can change the effective line width, surface resistance, and resonant frequency. Therefore, embroidered metamaterial antennas should be evaluated together with fabrication tolerance, bending response, washing durability, and repeatability [84,85,87,88,89,90].

### 4.3. Printing and Coating

Screen-printed silver ink, conductive paste, resistive films, carbon-based coatings, and MXene coatings have been used to form resonant or lossy textile patterns [18,34,36,79,91]. Table 9 summarizes representative printed and coated metamaterial structures, including silver-ink textile absorbers, printed FSS patterns, inkjet-printed absorbers, MXene-based metasurfaces, and coated nonwoven fabrics. These studies show that printing and coating are not only patterning methods, but also material-tuning routes for controlling conductivity, sheet resistance, and absorption loss.

The key challenge is the interaction between ink and fabric. Ink spreading, penetration into yarn gaps, uneven drying, and cracking under bending can change line width, thickness, and surface resistance. These variations directly affect the resonant frequency, absorption strength, and frequency-selective response. Therefore, printed textile metamaterials should be evaluated together with sheet resistance stability, bending durability, washing resistance, and pattern repeatability.

### 4.4. Laser Patterning and Etching

Laser patterning and etching provide better geometric precision than manual cutting or coarse coating. They are suitable for fine patterns, especially when the operating frequency is high, and the unit-cell size is small [15,18,28]. Table 10 summarizes representative laser-patterned or etched structures, including etched copper-laminate EBGs, laser-cut conductive textile metasurfaces, laser-induced graphene absorbers, textile CSRR cells, and laser-written antenna patterns. These examples indicate that laser processing can improve edge definition and pattern repeatability, but its use depends on substrate thermal stability.

Overall, laser patterning and etching offer relatively high geometric resolution for textile metamaterials, especially for fine patterns. Compared with manual cutting or coarse coating, these methods improve edge definition and pattern repeatability, which is important for unit cells sensitive to dimensional errors. However, their textile application is limited by substrate thermal stability, processing cost, material compatibility, and possible fabric damage. Therefore, laser-based techniques are suitable for precise patterning and mechanism verification, but practical integration still requires control of thermal effects, sheet resistance, adhesion, and mechanical durability [94,95].

## 5. Reliability of Textile Metamaterial

### 5.1. Bending and Deformation Stability

**Figure 5:**
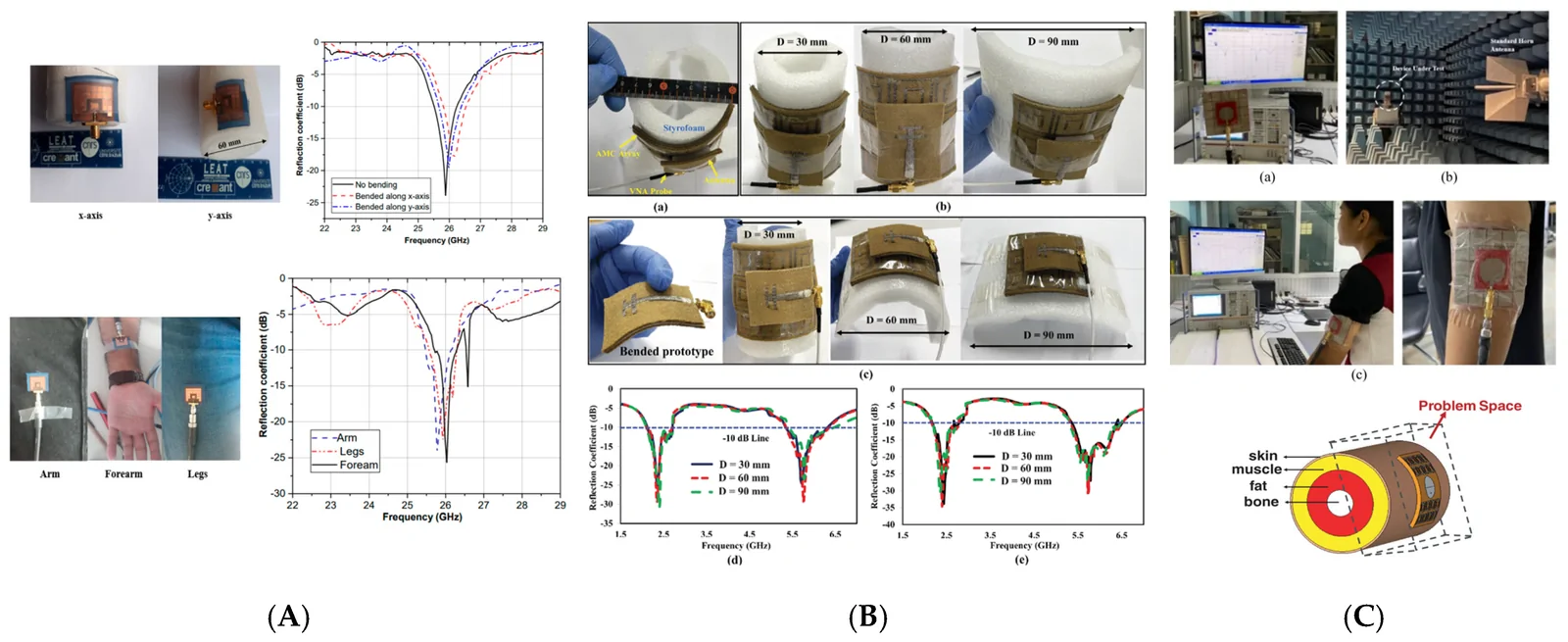

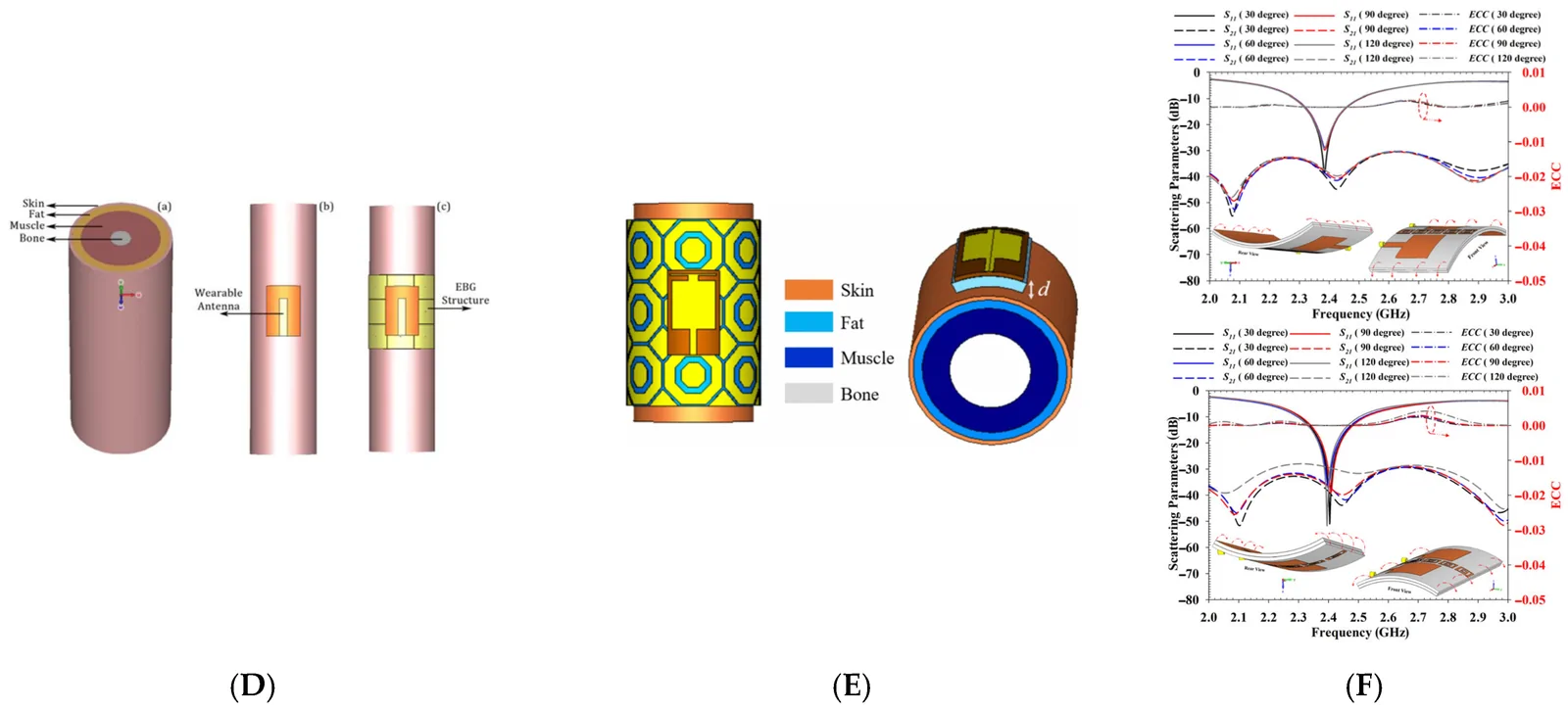
Representative bending, on-body, and directional deformation evaluations of textile metamaterial-integrated antennas: (**A**) [15] Wissem; (**B**) [12] Ali; (**C**) [45] Thaiwirot; (**D**) [96] Tetik; (**E**) [46] Hengroemyat; (**F**) [58] Agus.

The wearability of textile metamaterial-integrated antennas is commonly evaluated through static bending and on-body tests. As shown in Figure 5, the antenna is usually mounted on cylindrical supports with different diameters or placed on-body locations such as the arm, forearm, and leg, and its performance is compared before and after deformation. Wissem et al. [15] found that a millimeter-wave EBG antenna retained its target operating band under different bending directions and body positions, although some resonance shift occurred. Ali et al. [12] tested a dual-band AMC antenna on cylinders with diameters of 30, 60, and 90 mm. The operating bands were generally maintained, while smaller bending diameters caused more evident detuning. The UWB AMC antenna reported by Thaiwirot et al. [45] preserved its wideband response under arm-wrapping and on-body conditions, with only a slight shift in the lower-frequency region. Hengroemyat et al. [46] further evaluated antenna stability near the body using a multilayer arm model.

Bending direction also affects antenna performance. Shamsuri Agus et al. [58] examined an RIS–EBG MIMO antenna under different bending angles and orientations. The scattering parameters and mutual coupling varied differently along the two axes, indicating clear directional sensitivity. This behavior was associated with changes in unit-cell spacing, current paths, and relative array positions.

Overall, existing studies confirm that these antennas can maintain acceptable performance under regular bending and static on-body conditions. However, the reported tests remain relatively simple. Wrinkling, twisting, nonuniform compression, interlayer sliding, localized severe deformation, and dynamic body motion have rarely been evaluated systematically. Future work should therefore include irregular deformation and dynamic wearing tests.

### 5.2. Moisture, Washing, Abrasion, and Long-Term Durability

Textile substrates are porous and moisture-sensitive. Water absorbed into the fibers and interlayer voids can increase the effective permittivity and dielectric loss, while also altering the coupling between metamaterial cells and between the antenna and the AMC layer. Kamardin et al. evaluated textile diamond-dipole, CPW-monopole, and textile-AMC configurations under wet conditions [97,98,99,100]. After prolonged immersion, the fully wet structures exhibited clear downward frequency shifts and distorted responses. Their performance gradually recovered during drying, although small residual deviations remained, possibly due to fabric shrinkage, interlayer displacement, or residual moisture. These studies mainly examined single-immersion and drying-recovery processes and should not be regarded as standardized laundering tests.

**Figure 6:**
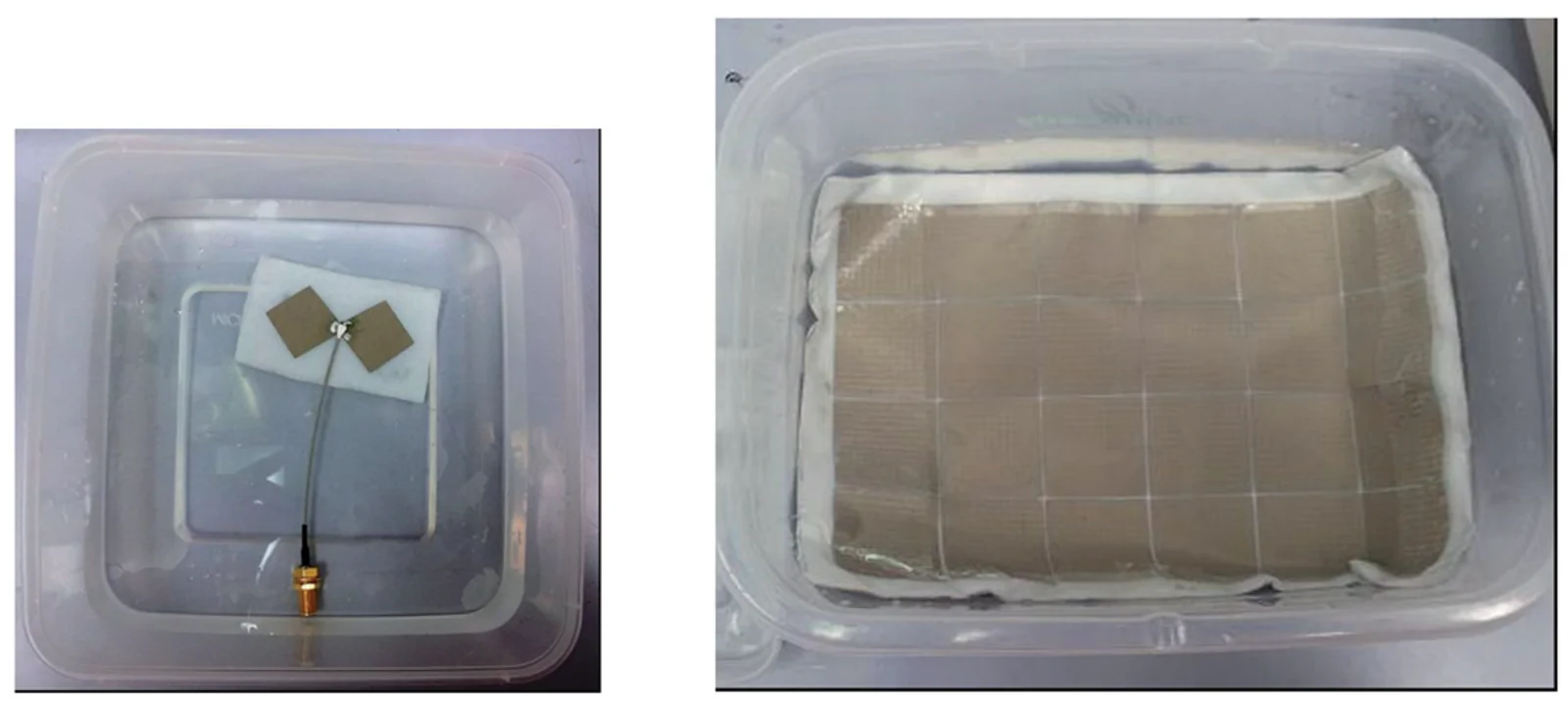
Wetness-test configurations for textile AMC [98].

Existing evaluations also focused largely on S11, with limited systematic comparisons of the AMC reflection phase, gain, efficiency, radiation pattern, and SAR before and after wetting. Standardized abrasion tests, artificial-sweat exposure, repeated wet–dry cycling, and post-washing performance assessment remain insufficiently investigated for textile metamaterial-integrated antennas (Figure 6).

## 6. Conclusions and Future Perspectives

### 6.1. Conclusions

Metamaterials provide an effective route for improving the on-body performance of wearable antennas by introducing additional electromagnetic interfaces to regulate antenna–body coupling, suppress backward radiation, enhance radiation efficiency, reduce SAR, and improve polarization response. Among the reported structures, AMC/HIS and EBG are most frequently used for reducing backward radiation, improving gain, increasing front-to-back ratio, and lowering SAR. FSS provide more selective transmission, reflection, shielding, beam shaping, and polarization control. Resonator-loaded structures are useful for compact and multiband textile antennas. These functions are often coupled in practical textile antenna stacks, so the role of a metamaterial layer should be evaluated according to its position, spacing, coupling strength, and interaction with the radiator and human body.

The reviewed studies also show that textile is a decisive part of metamaterial antenna design. Substrate permittivity, loss tangent, thickness, spacer configuration, conductor sheet resistance, pattern accuracy, and layer alignment directly affect the final reflection phase, band-gap response, frequency selectivity, resonant mode, radiation efficiency, and SAR performance. Therefore, textile substrates, spacer layers, and conductive materials should not be treated only as mechanical supports. They are active design variables that determine whether the intended metamaterial response can be transferred from simulation to a wearable textile platform.

As for fabrication, lamination is suitable for low-cost and rapid laboratory validation of designs, but its accuracy and long-term reliability are limited by cutting tolerance, adhesive thickness, bonding uniformity, and layer misalignment. Embroidery, sewing, weaving, and knitting offer better textile integration, but their electromagnetic response is affected by yarn resistance, stitch geometry, loop deformation, and structural repeatability. Printing and coating allow conductivity or surface resistance to be adjusted, whereas ink spreading, cracking, adhesion, and bending stability remain important concerns. Laser patterning and etching can improve edge definition and pattern resolution, but textile application is still restricted by thermal stability, material compatibility, and processing cost.

Future research could place greater emphasis on the design of metamaterial structures tailored to textile material characteristics. Metamaterials should be evaluated together with antenna matching, gain, efficiency, radiation pattern, AR, SAR, on-body detuning, and fabrication repeatability. For practical garment integration, greater attention must be paid to layer spacing, conductor continuity, bonding stability, connector transition, flexibility, breathability, and long-term wearing reliability. A practical textile metamaterial antenna should balance electromagnetic performance, textile compatibility, manufacturability, and human-body safety. With the advances in artificial intelligence, more intelligent and efficient design frameworks could also be established to support material selection, structural optimization, performance prediction, and parameter screening for textile metamaterial antennas.

### 6.2. Future Perspectives

Further development should move beyond transferring conventional resonators onto fabric and instead integrate electromagnetic functions into the textile structure. Conductive-polymer fabric antennas and stretchable textile antennas have shown that radiation and sensing functions can be incorporated into soft and deformable platforms [101,102,103,104]. Similar approaches could be extended to textile metamaterials by combining weaving, knitting, embroidery, printing, or additive manufacturing to form the radiator, periodic cells, conductive paths, and protective layers within a unified textile system. The main challenge is to retain unit-cell geometry, sheet resistance, and layer spacing during bending, stretching, washing, and repeated wear. Conductive-polymer fabric antennas have already demonstrated the feasibility of fully organic RF structures, while stretchable textile antennas indicate how mechanical deformation and electromagnetic response may be jointly designed.

Material and structural concepts developed outside textile antennas also provide useful references. Graphene and carbon films offer a basis for thin and lightweight passive components across the microwave–THz range [105]. Graphene split-ring structures further show that material tunability and geometric asymmetry can be combined to obtain multiband and polarization-dependent responses [106]. These principles may support future textile metasurfaces based on coated fibers, conductive films, or hybrid carbon-textile layers, although their conductivity, biasing method, adhesion, and durability still require experimental verification on fabrics. Textile rectenna arrays and metamaterial-assisted rectennas further demonstrate the potential to combine communication with RF energy harvesting [107,108]. Reflective textile metasurfaces can also support indoor signal-coverage enhancement [109]. The cited graphene absorber achieved tunable multiband and strongly anisotropic responses through a single patterned resonator layer, but its THz-scale rigid multilayer configuration cannot be transferred directly to clothing systems.

Compact rigid antennas may also provide structural guidance. Ground-integrated CSRR loading has been used to lower the resonant frequency and reduce antenna size in a 5G design [110]. However, the reported negative gain also illustrates that miniaturization can be accompanied by reduced radiation performance. Textile adaptation should therefore evaluate size reduction together with gain, efficiency, bandwidth, conductor loss, and deformation stability rather than reproducing the rigid geometry directly. The cited design reduced the operating frequency from 5.88 to 3.5 GHz through CSRR loading and reported substantial miniaturization, but its simulated gain was −3.7 dBi.

Programmable surfaces and data-driven design provide longer-term opportunities. Field-programmable RF surfaces have demonstrated real-time regulation of current paths, impedance matching, and radiation characteristics on conventional substrates [111,112]. Their textile implementation would require flexible low-loss switches, reliable bias networks, compact power supplies, and washable encapsulation. Fully textile programmable metasurfaces and reconfigurable intelligent surfaces therefore remain exploratory rather than established solutions. Machine learning has also been applied to wearable-antenna optimization [113]. Future models should include textile-specific variables, such as sheet resistance, anisotropy, moisture, compression, bending radius, layer misalignment, and fabrication tolerance, and jointly consider gain, bandwidth, efficiency, SAR, size, flexibility, and reliability.

## Figures and Tables

**Table 1:** Representative AMC/HIS configurations and performance comparison of wearable textile antennas with and without AMC/HIS integration.

Ref.	Structure	Bandwidth	Gain	Efficiency	SAR
[11] Yan	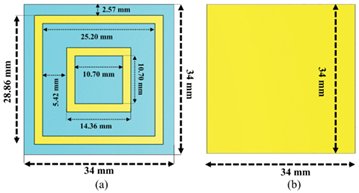	-	-	-	-
[12] Ali	3 × 3 dual-band AMC array 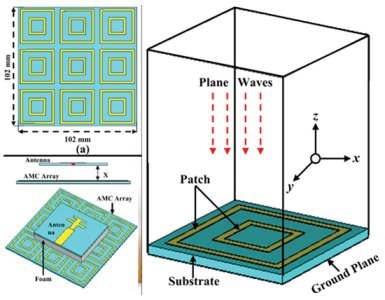	Sim., on-body: 2.45 GHz: 326 → 430 MHz 5.8 GHz: 792 → 1001 MHz Meas., on-body: 2.45 GHz: 720 → 472 MHz 5.8 GHz: 425 → 1013 MHz	Sim.: 2.45 GHz: −6.09 → 8.55 dBi 5.8 GHz: 1.65 → 4.31 dBi Meas.: 2.45 GHz: −7.17 → 7.83 dBi 5.8 GHz: 2.64 → 4.43 dBi	Sim.: 2.45 GHz: 45.54% → 81.83% 5.8 GHz: 53.57% → 73.14% Meas.: 2.45 GHz: 43.55% → 78.71% 5.8 GHz: 55.20% → 68.18%	Sim., Pin = 0.5 W: 2.45 GHz, 1 g: 11.5 → 0.138 W/kg 2.45 GHz, 10 g: 5.05 → 0.0669 W/kg 5.8 GHz, 1 g: 4.27 → 0.0521 W/kg 5.8 GHz, 10 g: 1.58 → 0.0322 W/kg
[48] Atrash	AMC-backed monopole 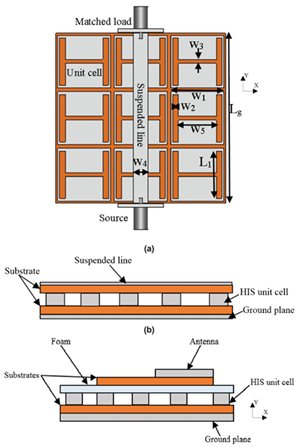	-	-	-	-
[43] Ashyap	HIS	-	1.96 → 7.47 dBi	-	Sim., 2.45 GHz, 1 g, Pin = 0.1 W, direct contact: Chest: 7.80 → 0.133 W/kg Arm: 10.30 → 0.069 W/kg
[44] Ashyap	AMC zero-phase shield	-	On chest: −9 → 6.21 dB	On chest: 10% → 81%	-
[54] Giman	Dual-band AMC 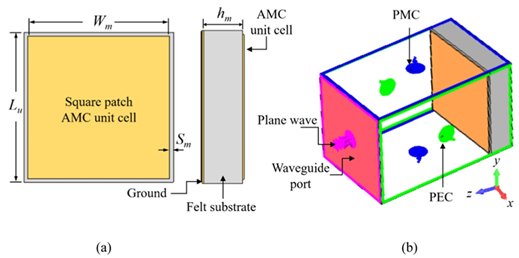	-	-	-	-
[45] Thaiwirot	Low-profile AMC 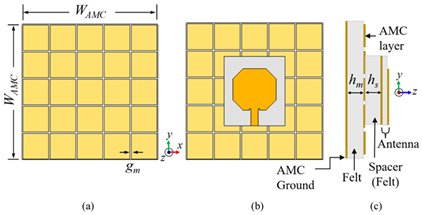	-	Sim., across the operating band: 1.52–4.18 → 3.38–8.57 dBi	-	Sim., 5.2 GHz, 10 g, Pin = 0.5 W: 22.9 → 0.362 W/kg
[46] Hengroemyat	Dual-band AMC 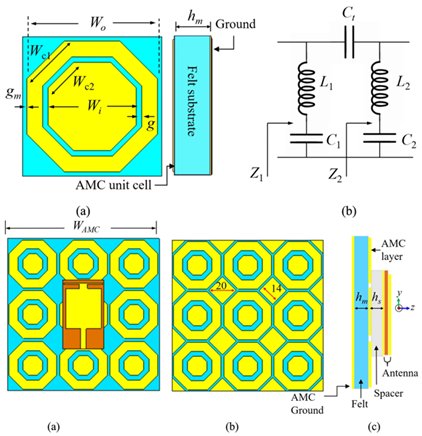	Meas.: Lower band: 1.88–3.55 → 2.04–2.63 GHz Upper band: 4.88–6.73 → 5.05–5.48 GHz	Sim.: 2.4 GHz: 2.50 → 7.27 dBi 5.2 GHz: 3.17 → 5.94 dBi	-	Sim., 1 g, Pin = 0.5 W: 2.4 GHz: 23.57 → 0.467 W/kg 5.2 GHz: 29.20 → 0.205 W/kg
[50] Suwailam	HIS 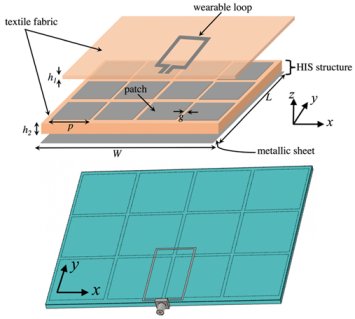	-	-	Meas.: Cotton: 89% → 80% Jeans: 77% → 55%	-
[51] Joshi	3 × 3 AMC 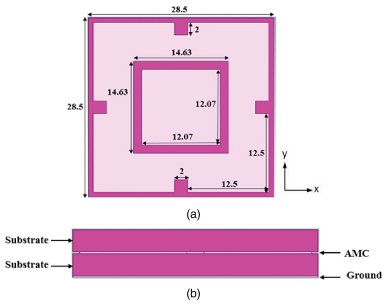	-	-	1.575 GHz: 48% → 73.6% 2.45 GHz: 45% → 83%	-
[53] Hussain	AMC	-	-	-	-
[55] Yalduz	UWB metamaterial reflector	-	-	-	Sim., 10 g: 4 GHz: 6.27 → 0.067 W/kg 7.5 GHz: 2.23 → 0.107 W/kg 10.5 GHz: 1.94 → 0.070 W/kg
[49] Atrash	Dual-band low-profile AMC	-	-	-	-
[56] Thangavelu	AMC 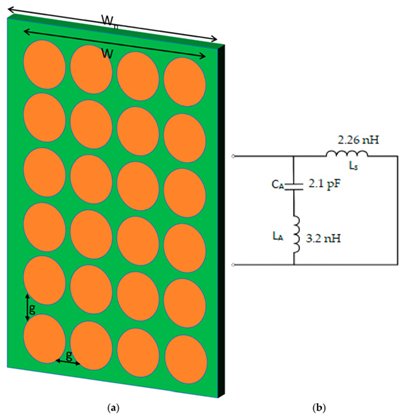	-	Sim., 2.45 GHz: Cotton: 5.72 → 7.09 dBi Jean: 5.56 → 6.72 dBi Jute: 5.56 → 6.76 dBi	-	-
[52] Yang	Dual-band dual CP AMC	-	-	-	-
[57] Casula	AMC 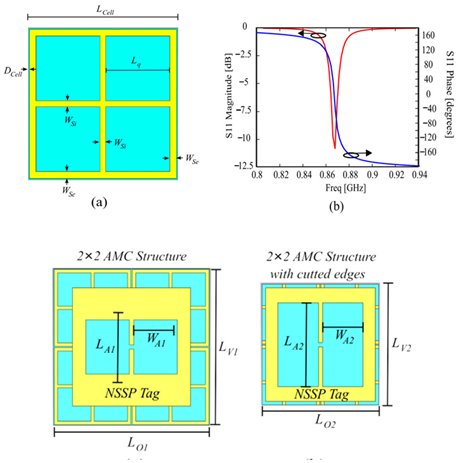	-	-	-	-

**Table 2:** Representative EBG configurations and performance comparison of wearable textile antennas with and without EBG integration.

Ref.	Structure	Bandwidth	Gain	Efficiency	SAR
[13] Zhu	Dual-band EBG/HIS	Meas.: Lower band: 17% → 4% Upper band: 16% → 12%	Meas.: 2.45 GHz: 3.9 → 6.4 dBi 5.8 GHz: 5.2 → 7.6 dBi		Separate 1.8 GHz SAR-validation antenna: Sim., 1 g: 11.47 → 0.48 W/kg Sim., 10 g: 7.18 → 0.31 W/kg Meas., 1 g: 12.87 → 0.90 W/kg Meas., 10 g: 7.96 → 0.42 W/kg
[14] Ashyap	Via-less EBG 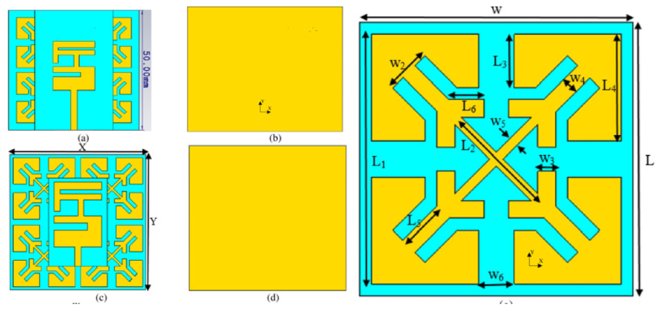		2.2 → 7.2 dBi		
[15] Wissem	Millimeter-wave EBG 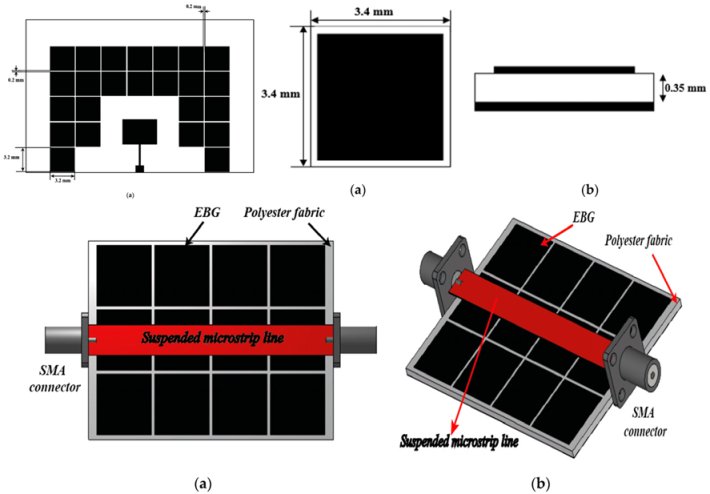	Meas.: 0.91 → 0.76 GHz	Sim.: 6.97 → 9.79 dBi Meas.: 6.13 → 8.65 dBi	Sim.: 72% → 77% Meas.: 54% → 61%	Sim., 26 GHz, 10 g, 1 mm: 0.32 → 0.096 W/kg
[58] Agus	RIS and EBG 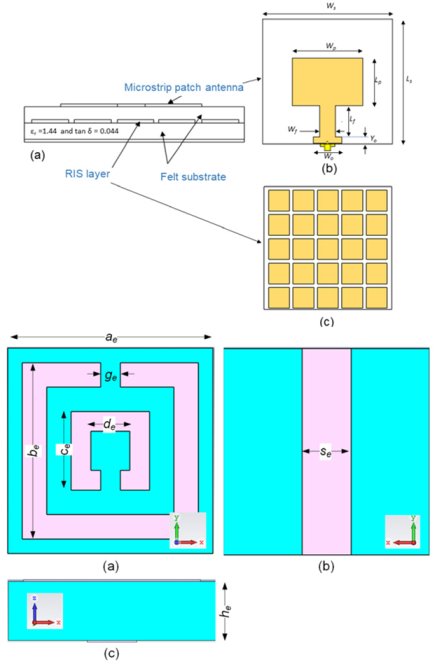				
[60] Tak	EBG				
[59] Kumkhet	Dual-band EBG				

**Table 3:** Representative FSS configurations and performance comparison of wearable textile antennas with and without FSS integration.

Ref.	Structure	Bandwidth	Gain	Efficiency	SAR
[17] Amit	FSS				
[18] Rumijowska	FSS 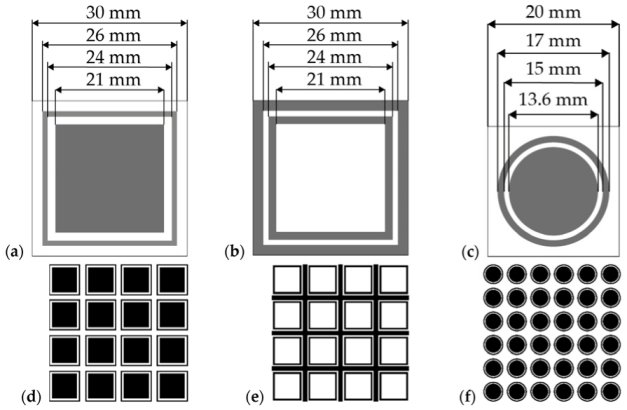				
[62] Sugumaran	square-ring FSS		Sim.: 2.56 → 8.20 dBi Meas.: 2.62 → 7.78 dBi	Sim.: 96.91% → 95.04% Meas.: 88.51% → 80.12%	Sim., 2.45 GHz, 1 g, Pin = 0.1 W, 1 mm: 3.197 → 0.164 W/kg
[63] Vaezi	FSS				
[21] Zheng	FSS				
[66] Guan	Ring-shaped FSS				
[67] Li	Y-shaped FSS				
[69] Zhu	FSS 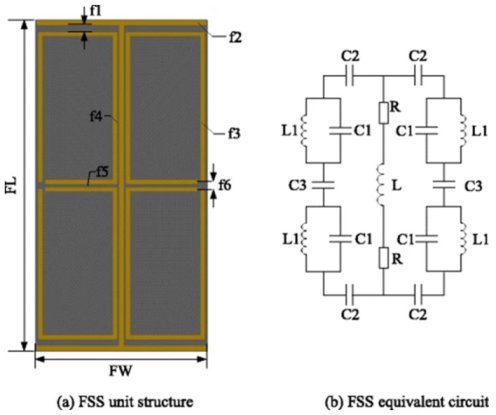				

**Table 4:** Comparison of AMC/HIS, EBG, and FSS structures in wearable textile antennas.

Structure Type	Typical Structure	Main Performance Contribution	Representative Applications
AMC/HIS	Periodic metal cells, dielectric substrate, ground; vias are optional	In-phase reflection, backward-radiation suppression, gain and F/B-ratio enhancement, and SAR reduction	Low-profile and low-SAR WBAN antennas, wearable healthcare devices, and off-body communication
EBG	Periodic metal cells on a dielectric substrate; the ground and vias depend on the topology	Surface-wave suppression or guidance, coupling reduction, gain enhancement, and improved on-body stability	Wearable MIMO decoupling, body-surface-wave communication, and millimeter-wave 5G-IoT systems
FSS	Periodic metal or aperture layer/dielectric substrate; generally without a continuous ground	Frequency-selective reflection and transmission, shielding, filtering, beam control, and gain enhancement	Frequency-selective fabrics, multiband wearable antennas, shielding layers, and FSS-based MIMO decoupling

**Table 5:** Materials and properties of substrates and spacer layers used in textile metamaterial structures.

Ref.	Layer Type	Materials	Thickness (mm)	ε_r_	Tanδ	Pic.
[13] Zhu	substrate		2.2	1.38	0.02	
	spacer materials	Rohacell	1.1	1.006		
[14] Ashyap	substrate		0.7	1.7		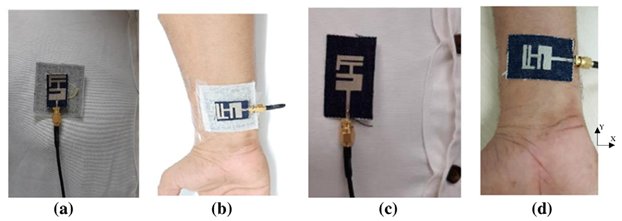
	spacer materials	foam	1			
[17] Amit	substrate	Flannel	1	1.7	0.0025	
	spacer materials	Air gap	1	1.0	0	
[28] Kim	radiator substrate	Felt	1	1.4	0.044	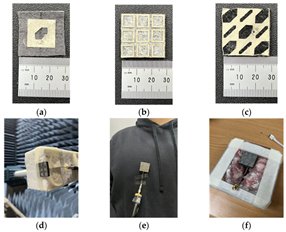
	metasurface substrate	Felt	4	1.4	0.044	
[11] Yan	substrate	Felt	1.5	1.2	0.044	
[12] Ali	substrate	Felt	2	1.3	0.044	
[35] Yang	spacer materials	Scuba knitting fabric	2.6–3.0	1.5–2.1		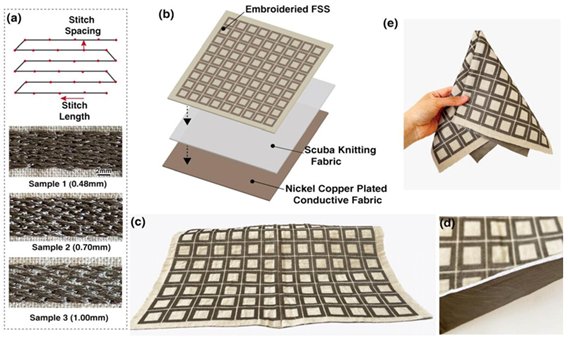
[75] Yang	encapsulation/host	Ecoflex elastomer	1.45			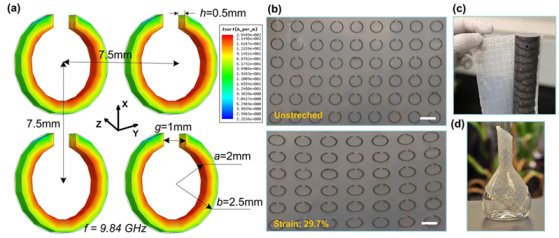
[76] Kim	substrate/channel layer	PDMS	1.3			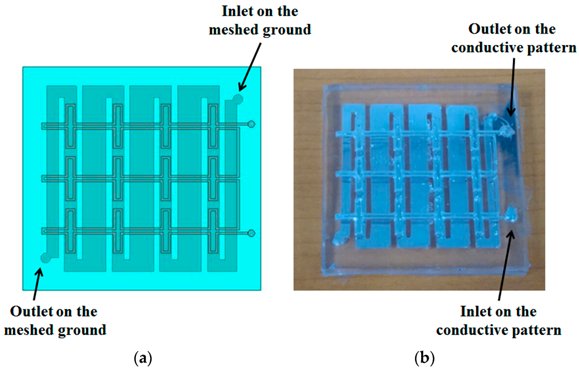
[48] El Atrash	spacer	Air gap	5	1.0	0	
[45] Thaiwirot	antenna substrate	Felt	1	1.4	0.04	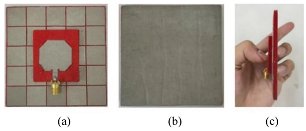
	AMC substrate	Felt	4	1.4	0.04	
[46] Hengroemyat	antenna substrate	Felt	2	1.4	0.04	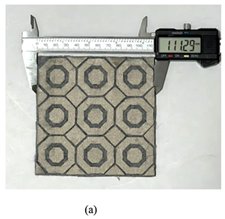
	AMC substrate	Felt	4	1.4	0.04	
	spacer materials	Flexible foam	4			
[18] Rac-Rumijowska	substrate	PET polyester textile	0.20	1.9		
	comparator substrate	PET foil	0.25	3.4		
[19] Cheng	substrate	Velvet/common textile substrate				
[58] Agus	substrate	Viscose-wool felt	3	1.44	0.044	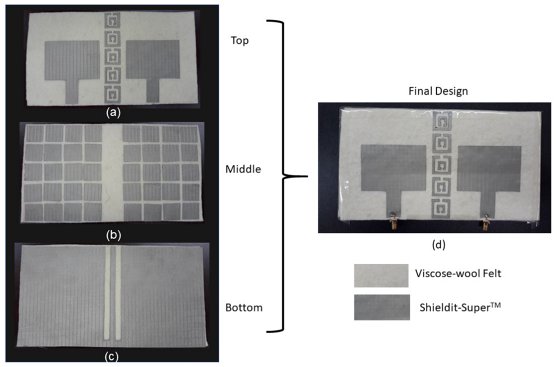
[77] Ramasamy	substrate	PDMS flexible substrate				
[27] Regina	substrate	Leather flexible substrate				
[56] Thangavelu	substrate	Cotton	0.8	1.6	0.04	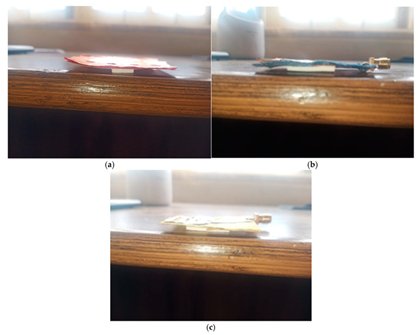
	substrate	Jean	1.1	1.7	0.025	
	substrate	Jute	1.2	1.87	0.052	
	spacer materials	PU foam	3			
	AMC equivalent substrate		1.8			

**Table 6:** Conductive materials and properties used in textile metamaterial structures.

Ref.	Thickness (mm)	Conductivity (S/m)	Material
[13] Zhu	0.06	1 × 10^6^	Zelt conducting material; high-quality nylon-based substrate plated with copper
[13] Zhu	0.17	1.18 × 10^5^	ShieldIt Super; ripstop woven polyester textile plated with copper and nickel
[14] Ashyap	0.17	1.18 × 10^5^	ShieldIt(TM) material used for the conducting elements
[15] Wissem		5.8 × 10^7^	Ultra-thin polyimide copper laminates; thin copper foil with adhesive backing
[28] Kim	0.17	118,000	Conductive textile used for the radiator, slot-patterned ground and metasurface
[11] Yan	0.17	1.18 × 10^5^	ShieldIt Super conductive textile from LessEMF Inc.
[12] Ali	0.17	1.18 × 10^5^	ShieldIt material plated with copper and nickel; surface resistivity < 0.05 Ohm/sq
[34] Lee	1.2 overall absorber thickness		Silver conductive ink screen printed on ordinary textile; copper tape ground plane
[35] Yang	0.70 stitch density		Embroidered frequency-selective surface (FSS), scuba knitting fabric and metalized fabric
[36] Yang	2.6-3.0 substrate thickness	45-100 Ohm/sq	Commercial PU conductive films used as resistive film patterns; metalized fabric ground plane
[75] Yang	0.5	3.4 × 10^6^	EGaIn liquid-metal split-ring resonators embedded in Ecoflex elastomer
[76] Kim	0.7 top-channel height; 1.0 bottom-channel height	3.4 × 10^6^	EGaIn liquid metal injected into PDMS microfluidic channels
[78] Celenk		<100 Ohm/m	Madeira HC-12 silver-coated conductive thread embroidered on textile materials
[45] Thaiwirot	0.17	1.18 × 10^5^	Copper-nickel plated polyester fabric used for the conductive parts
[46] Hengroemyat	0.17	1.18 × 10^5^	Copper-nickel plated polyester fabric used for the radiating and ground elements
[18] Rac-Rumijowska	0.025 catalog layer thickness	about 50 mOhm/sq	DuPont Intexar PE874 conductive screen-printing paste on PET textile
[37] Kim	0.035 copper layers	5.8 × 10^7^	Patterned copper, continuous copper layer and polypyrrole conductive fibers
[79] Zhang	3.17 calcium alginate/MXene; 1.36 cotton/MXene		Ti3C2TX MXene coated on polyester, cotton and calcium-alginate non-woven fabrics
[80] Jung	0.2 printing layer; 4 SRR thickness		Carbon-black-reinforced PLA conductive filament printed onto conductive textile substrate

**Table 7:** Representative textile metamaterial antennas fabricated by lamination.

Ref.	Fabrication Method	Pics
[43] Ashyap	Patterned conductive-fabric HIS laminated on textile substrate	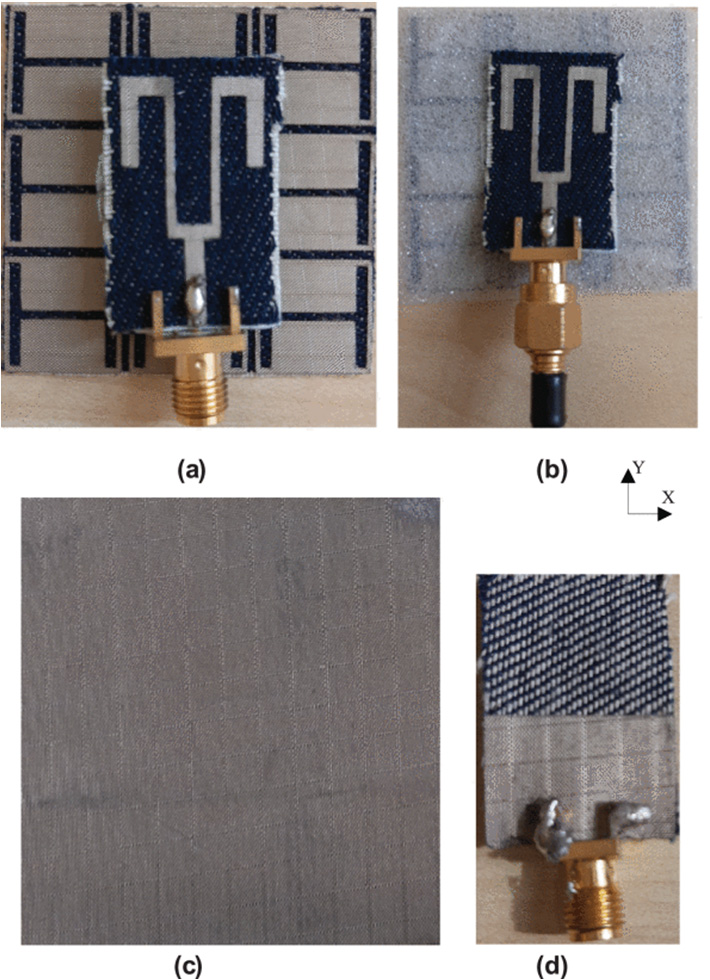
[45] Thaiwirot	Cut Cu-Ni plated fabric laminated with felt layers	
[46] Hengroemyat	Octagonal Cu-Ni plated fabric cells laminated on a felt/foam stack	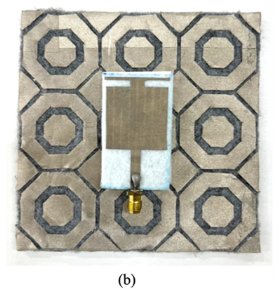
[50] Bait-Suwailam	Textile HIS laminated with a grounded loop antenna	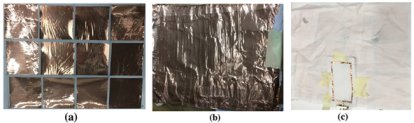
[51] Joshi	Square-patch AMC backing laminated with a textile antenna	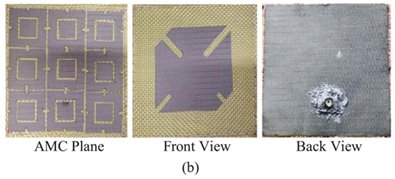
[81] Hossain		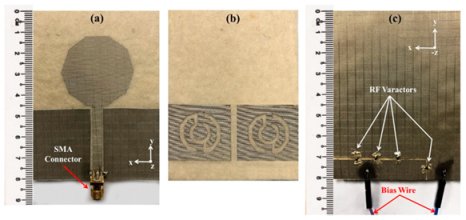
[82] Ali	Metamaterial-inspired conductive pattern laminated on flexible substrate	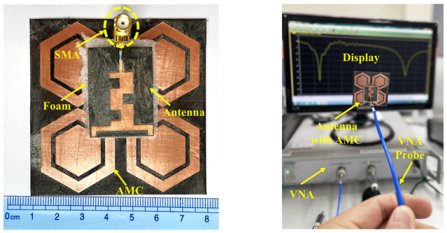

**Table 8:** Representative textile metamaterial antennas fabricated by embroidery and sewing.

Ref.	Fabrication Method	Pics
[12] Ali	Conductive textile/stitched layers assembled into a dual-band AMC antenna	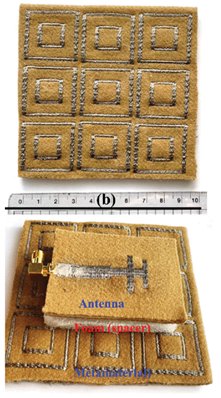
[35] Yang	Embroidered FSS pattern on scuba fabric with metalized backing	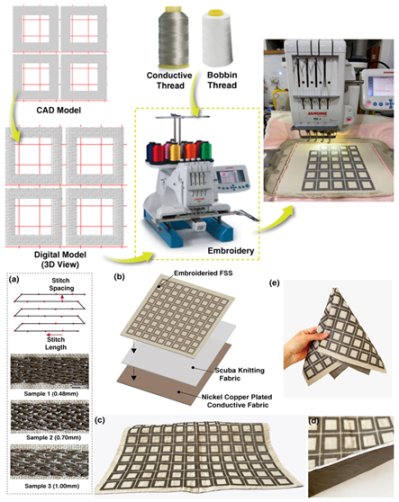
[78] Celenk ^1^	Silver-coated thread embroidery of SRR arrays on textile/polyester layers	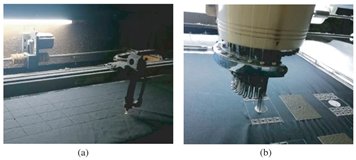
[86] Zhu	Digital embroidery of textile ducts for coaxial metamaterial resonators	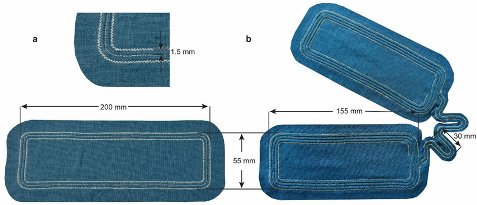

^1^ The images in the row of Ref. [78] are reproduced courtesy of The Electromagnetics Academy.

**Table 9:** Representative textile metamaterial antennas fabricated by printing and coating.

Ref.	Fabrication Method	Pics.
[34] Lee	Screen-printed silver-ink absorber pattern on textile	
[18] Rac-Rumijowska	Screen-printed silver-paste FSS on PET textile/film	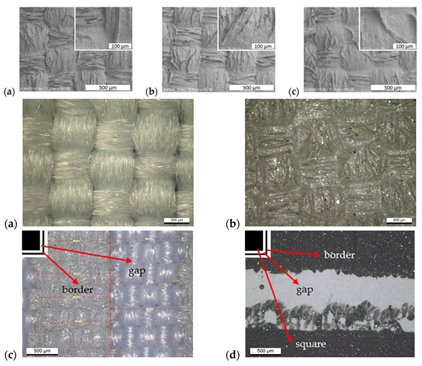
[92] Kim	Inkjet-printed resonant absorber pattern on flexible substrate	
[93] Ling	Inkjet-printed absorber integrated with microfluidic tuning	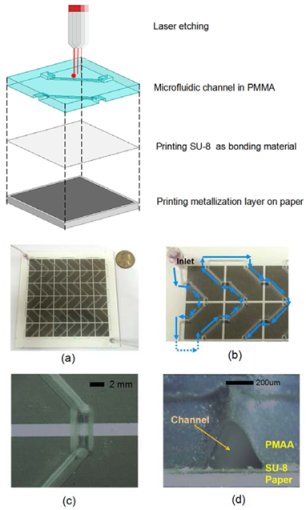
[91] Huang	Inkjet-printed MXene metasurface pattern	
[79] Zhang	MXene-coated nonwoven fabric laminate	

**Table 10:** Representative textile metamaterial antennas fabricated by laser patterning and etching.

Ref.	Fabrication Method	Pics.
[15] Wissem	Etched polyimide copper laminate with adhesive copper layers	
[28] Kim	Laser-cut conductive textile/felt layers for patch, ground, and metasurface	
[94] Chen	Laser-induced graphene/Fe_3_O_4_ metasurface patterning	
[26] Hossain	Fabric-laminated textile CSRR unit for microwave characterization	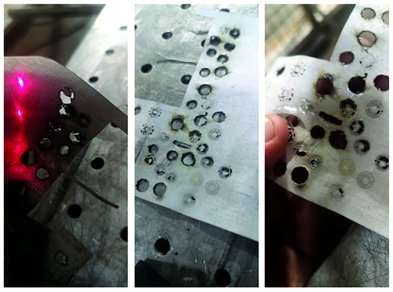
[95] Sartanavicius	Laser-induced graphene antenna pattern on polyimide	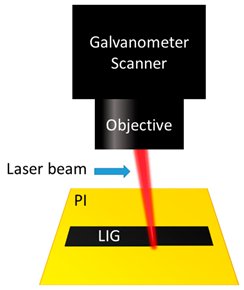

## Data Availability

No new data were created or analyzed in this study. Data sharing is not applicable to this article.

## Abbreviations

3D	Three-Dimensional
5G	Fifth-Generation Mobile Communication
AI	Artificial Intelligence
AMC	Artificial Magnetic Conductor
AR	Axial Ratio
CP	Circular Polarization
CPW	Coplanar Waveguide
CRLH	Composite Right/Left-Handed
CSRR	Complementary Split-Ring Resonator
EBG	Electromagnetic Band Gap
EGaIn	Eutectic Gallium–Indium
EMI	Electromagnetic Interference
F/B	Front-to-Back Ratio
FSS	Frequency-Selective Surface
GNSS	Global Navigation Satellite System
GPS	Global Positioning System
HIS	High-Impedance Surface
IoT	Internet of Things
MIMO	Multiple-Input Multiple-Output
MXene	Transition Metal Carbide, Nitride, or Carbonitride
PDMS	Polydimethylsiloxane
PEC	Perfect Electric Conductor
PET	Polyethylene Terephthalate
PIN	Positive–Intrinsic–Negative
PLA	Polylactic Acid
PU	Polyurethane
RF	Radio Frequency
RFID	Radio-Frequency Identification
RIS	Reconfigurable Intelligent Surface
SAR	Specific Absorption Rate
SIW	Substrate-Integrated Waveguide
SRR	Split-Ring Resonator
UHF	Ultra-High Frequency
UWB	Ultra-Wideband
WBAN	Wireless Body Area Network
WLAN	Wireless Local Area Network
ZOR	Zeroth-Order Resonator
